# Development and External Validation of a Nomogram to Predict Recurrence-Free Survival After R0 Resection for Stage II/III Gastric Cancer: An International Multicenter Study

**DOI:** 10.3389/fonc.2020.574611

**Published:** 2020-10-22

**Authors:** Jun Lu, Bin-bin Xu, Chao-hui Zheng, Ping Li, Jian-wei Xie, Jia-bin Wang, Jian-xian Lin, Qi-yue Chen, Mark J. Truty, Chang-ming Huang

**Affiliations:** ^1^Department of Gastric Surgery, Fujian Medical University Union Hospital, Fuzhou, China; ^2^Department of General Surgery, Fujian Medical University Union Hospital, Fuzhou, China; ^3^Key Laboratory of Ministry of Education of Gastrointestinal Cancer, Fujian Medical University, Fuzhou, China; ^4^Fujian Key Laboratory of Tumor Microbiology, Fujian Medical University, Fuzhou, China; ^5^Department of Surgery, Mayo Clinic, Rochester, MN, United States

**Keywords:** gastric cancer, recurrence patterns, adjuvant chemotherapy benefit, nomogram, web-based tool

## Abstract

**Background:** The benefit of adjuvant chemotherapy varies widely among patients with stage II/III gastric cancer (GC), and tools predicting outcomes for this patient subset are lacking. We aimed to develop and validate a nomogram to predict recurrence-free survival (RFS) and the benefits of adjuvant chemotherapy after radical resection in patients with stage II/III GC.

**Methods:** Data on patients with stage II/III GC who underwent R0 resection from January 2010 to August 2014 at Fujian Medical University Union Hospital (FMUUH) (*n* = 1,240; training cohort) were analyzed by Cox regression to identify independent prognostic factors for RFS. A nomogram including these factors was internally and externally validated in FMUUH (*n* = 306) and a US cohort (*n* = 111), respectively.

**Results:** The multivariable analysis identified age, differentiation, tumor size, number of examined lymph nodes, pT stage, pN stage, and adjuvant chemotherapy as associated with RFS. A nomogram including the above 7 factors was significantly more accurate in predicting RFS compared with the 8^th^ AJCC-TNM staging system for patients in the training cohort. The risk of peritoneal metastasis was higher and survival after recurrence was significantly worse among patients calculated by the nomogram to be at high risk than those at low risk. The nomogram's predictive performance was confirmed in both the internal and external validation cohorts.

**Conclusion:** A novel nomogram is available as a web-based tool and accurately predicts long-term RFS for GC after radical resection. The tool can also be used to determine the benefit of adjuvant chemotherapy by comparing scores with and without this intervention.

## Introduction

Gastric cancer (GC) is the fifth most common malignancy worldwide and ranks third in cancer-related mortality (1). Radical gastrectomy is still the main treatment. However, even with radical resection, postoperative recurrence is common, affecting ~18 to 45.5% of patients (2–5). Patients with stage I GC have good prognosis and low recurrence rates, while outcomes for those with stage II/III GC patients vary widely and can be challenging to predict (3, 6–8).

Postoperative recurrence is the leading cause of death among patients with stage II/III GC (2, 8). Currently, the most widely used system for estimating survival and risk of recurrence is the 8th edition of the American Joint Committee on Cancer (AJCC) tumor-node-metastasis (TNM) staging system. However, for patients with non-metastatic GC, the AJCC-TNM system considers only two variables (pT, pN), and its ability to predict recurrence is still limited (2, 9). A predictive model including 6 variables that significantly better predicts overall survival (OS) than the AJCC-TNM staging system was developed by Han et al. but its predictive power for recurrence in patients with stage II/III GC was not evaluated (10). To better plan follow-up and treatment strategies for these patients, an individualized predictive tool to predict recurrence would be of value.

Another challenge in treating patients with stage II/III GC is determining who will benefit from adjuvant (postoperative) chemotherapy. While this treatment strategy has become standard for these patients (11–13) and has improved their long-term prognosis, whether all patients with stage II/III GC need adjuvant chemotherapy has been questioned (14, 15). A model to predict its benefit in patients with stage II/III GC was introduced by Jiang et al. but its predictive performance was not ideal (concordance index was 0.686), and it was not externally validated using Western data (16).

Therefore, in the present study, we used data from a large-volume center in China to construct a nomogram that can effectively predict postoperative recurrence and chemotherapy benefit in patients with stage II/III GC after radical surgery. The model was internally and externally validated using data from our center and a Western cohort, respectively. This is the first predictive model, which is available as a web-based tool, for postoperative recurrence and chemotherapy benefits based on international, multicenter data.

## Materials and Methods

### Datasets

The database at Fujian Medical University Union Hospital (FMUUH) was reviewed following approval from the Institutional Review Board (IRB). The inclusion criteria were as follows: histologically confirmed primary gastric cancer, no distant metastasis, and R0 gastrectomy performed between January 2010 and August 2014. The exclusion criteria included receiving neoadjuvant chemotherapy, pathologic stage I disease according to the 8^th^-AJCC-TNM staging system, remnant gastric cancer, and postoperative death within 3 months.

To examine the generalizability of the model, data on patients that satisfied the aforementioned inclusion and exclusion criteria were obtained from FMUUH between September 2014 and August 2015 (internal validation) and from the Mayo Clinic between January 2005 and December 2012 (external validation) following IRB approval.

Tumor stage, including pT, pN, and final stage, was determined according to the 8^th^ AJCC classification system (17).

### Follow-Up and Treatment

Follow-up visits for both cohorts generally consist of clinic visits every 3 months for the first 2 years and every 6 months for years 3 to 5. Most routine patient follow-up appointments include a physical examination, laboratory tests, chest radiography, abdominal ultrasonography, or CT, and an annual or biannual endoscopic examination for patients with a remnant stomach (18). Disease recurrence was diagnosed with radiologic findings on cross-sectional imaging or biopsies of suspicious lesions (3).

For those who could tolerate adjuvant chemotherapy, adjuvant chemotherapy was routinely recommended for patients with pathological stage II and III disease (19). The adjuvant chemotherapy consisted of either single-agent 5-fluorouracil (5-FU) or a combination of 5-FU and cisplatin/oxaliplatin or paclitaxel (19). To simplify of the nomogram, patients were classified as having received chemotherapy or not, regardless of the number of cycles (16).

### Definition and Categorization of Recurrence

Recurrences were categorized by site involved as previously described: (2, 3, 20) locoregional, peritoneal, distant, or multiple. Multiple recurrences were defined as the presence of recurrent disease in 2 or more sites. Early recurrence was defined as recurrence occurring within 12 months (3). Patients for which the exact site or sites of recurrence were unknown because of diagnosis in other hospitals were excluded from the analysis of recurrence patterns.

### Nomogram Construction, Validation, and Calibration

A Cox proportional hazards regression model was used to identify independent prognostic factors associated with RFS. Variables with *p* < 0.05 in the univariable analysis were subsequently included in the multivariable analysis, from which a nomogram was formulated in R for predicting the probability of 5-year recurrence-free survival (RFS).

The nomogram was subjected to 1,000 bootstrap resamples for internal and external validation. Its performance in predicting outcomes was evaluated by calculating the concordance index (C-index), area under the curve (AUC), and Akaike information criterion (AIC) (3). The nomogram was calibrated by comparing predicted with observed RFS after bias correction.

### Statistical Analysis

Continuous variables are reported as means ± SD or medians (interquartile ranges). Categorical variables were compared using the χ (2) or Fisher's exact test and continuous variables by *t*-test. RFS was assessed using the Kaplan–Meier method. Post-recurrence survival was defined as the period from the date of recurrence to the date of death or final follow-up. The non-linear relationship between nomogram-derived scores and RFS was modeled using restricted cubic splines (21). The nomogram's clinical usefulness was evaluated by decision curve analysis, which calculates the rate of true and false positives for various risk thresholds (3). The cohort was dichotomized into low-risk and high-risk subgroups by the median nomogram score among patients in the training cohort. Statistical analyses were performed using SPSS v.18.0 for Windows (SPSS Inc., Chicago, IL, USA) and R (https://www.r-project.org/). The R package “DynNom” was used to develop the web-based nomogram. *P* < 0.05 were considered statistically significant.

## Results

### Characteristics of the Training, Internal Validation, and External Validation Cohorts

A total of 1,240 patients with stage II/III GC who underwent radical gastrectomy were included in the training cohort. There were 306 and 111 patients were included in the internal and external validation cohort, respectively (Supplementary Figure 1). Table 1 shows the clinical and pathological data of both cohorts. The mean age of the training cohort was significantly younger than the external validation cohort (61.5 ± 11.2 vs. 68.3 ± 15.8 years), and included a higher proportion of male patients (75.5 vs. 56.8%). Adjuvant chemotherapy was administered to more patients in the training cohort (76.8 vs. 36.0%). The baseline characteristics was balanced between the training and internal validation cohorts.

**Table 1:** Patient and tumor characteristics in the training and validation cohorts.

	**Training cohort, no. (%)**	**Internal validation cohort, no. (%)**	***p*-value**	**External validation cohort, no. (%)**	*p*-value^*^
	FMUUH, *n* = 1240	FMUUH, *n* = 306		Mayo clinic, *n* = 111	
Age, mean years (SD)	61.5 (11.2)	60.4 (11.9)	0.147	68.3 (15.8)	<0.001
Tumor size, mean mm (SD)	51.1 (24.2)	52.4 (25.5)	0.397	67.0 (37.3)	<0.001
Examined LNs, mean No (SD)	36.9 (13.6)	36.9 (14.6)	0.999	25.2 (15.6)	<0.001
Sex			0.127		<0.001
Female	304 (24.5)	88 (28.8)		48 (43.2)	
Male	936 (75.5)	218 (71.2)		63 (56.8)	
Tumor location			0.801		0.011
Lower	474 (38.2)	120 (39.2)		42 (37.8)	
Middle	278 (22.4)	61 (19.9)		36 (32.4)	
Upper	316 (25.5)	79 (25.8)		28 (25.2)	
Mix	172 (13.9)	46 (15.0)		5 (4.5)	
Differentiation			0.910		<0.001
Well	56 (4.5)	14 (4.6)		0 (0.0)	
Moderate	446 (36.0)	106 (34.6)		11 (9.9)	
Poor	738 (59.5)	186 (60.8)		100 (90.1)	
Lymphovascular invasion^†^			0.493		NA
Absent	804 (64.8)	192 (62.7)		0 (0.0)	
Present	436 (35.2)	114 (37.3)		0 (0.0)	
Unknown	NA	NA		111 (100.0)	
Adjuvant chemotherapy			0.537		<0.001
Absent	288 (23.2)	66 (21.6)		71 (64.0)	
Present	952 (76.8)	240 (78.4)		40 (36.0)	
pT stage			0.659		0.003
T1	24 (1.9)	8 (2.6)		2 (1.8)	
T2	101 (8.1)	29 (9.5)		11 (9.9)	
T3	572 (46.1)	144 (47.1)		64 (57.7)	
T4a	515 (41.5)	121 (39.5)		27 (24.3)	
T4b	28 (2.3)	4 (1.3)		7 (6.3)	
pN stage			0.113		0.028
N0	195 (15.7)	43 (14.1)		30 (27.0)	
N1	233 (18.8)	49 (16.0)		23 (20.7)	
N2	285 (23.0)	92 (30.1)		20 (18.0)	
N3a	322 (26.0)	79 (25.8)		24 (21.6)	
N3b	205 (16.5)	43 (14.1)		14 (12.6)	
pTNM stage			0.493		0.031
II	804 (64.8)	192 (62.7)		49 (44.1)	
III	436 (35.2)	114 (37.3)		62 (55.9)	

*Continuous data represented as mean ± SD and categorical data as n (%)*.

* *Compared with the training cohort*.

† *Unavailable for the external validation cohort. FMUUH indicates Fujian Medical University Union Hospital; LN, lymph node; NA, not applicable*.

### Nomogram Development, Calibration and Internal Validation

**Figure 1:**
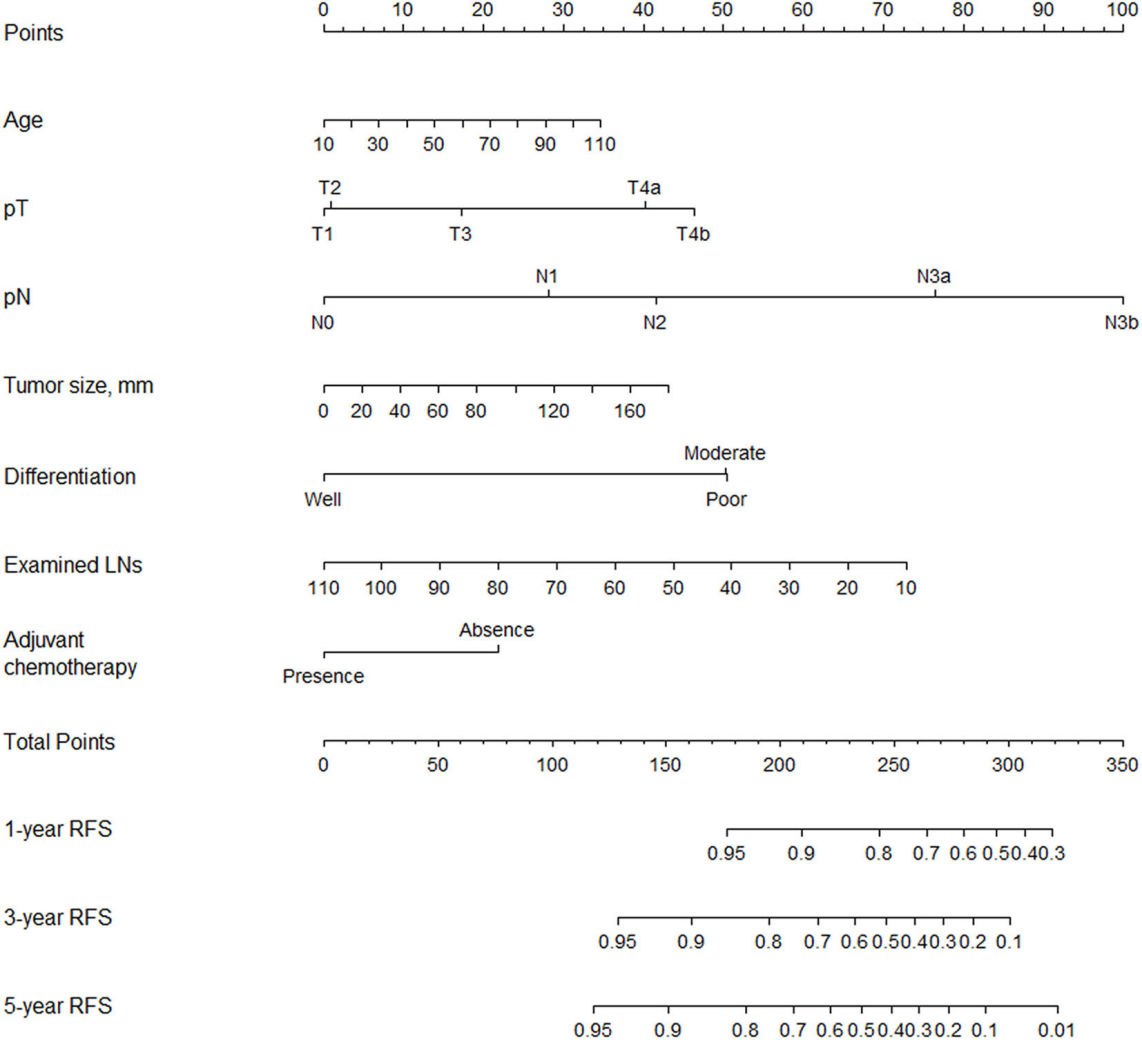
Nomogram for predicting 1-, 3-, and 5-year RFS after radical gastrectomy for stage II/III gastric adenocarcinoma.

Multivariable analysis identified age, differentiation, tumor size, number of examined lymph nodes, pT stage, pN stage, and adjuvant chemotherapy as associated with RFS (Table 2). We included the above variables in the predictive model to establish a nomogram and made it available online (https://qq406918430.shinyapps.io/DynamicPrediction/) (Figure 1, Supplementary Figure 2). The benefit of chemotherapy can be calculated by using the web-based calculating tool to determine the 5-year RFS probabilities both for the situation in which the patient receives or does not receive it; the difference between the two is the net survival benefit.

**Table 2:** Univariable and multivariable analyses of factors associated with recurrence-free survival.

	**Univariable**			**Multivariable**		
	**HR**	**95% CI**	***p*-value**	**HR**	**95% CI**	***p*-value**
Age	1.012	1.004–1.020	0.005	1.009	1.001–1.018	0.034
Tumor size	1.014	1.011–1.017	<0.001	1.005	1.002–1.009	0.003
Examined LNs	0.998	0.992–1.005	0.646	0.984	0.977–0.991	<0.001
Female sex vs. male	1.059	0.863–1.299	0.584			
Tumor location						
Lower	1.000			–		
Middle	1.258	0.993–1.592	0.057	–	–	0.446
Upper	1.069	0.845–1.353	0.578	–	–	0.892
Mix	1.660	1.280–2.152	<0.001	–	–	0.865
Histologic type						
Well	1.000			1.000		
Moderate	3.145	1.475–6.707	0.003	3.037	1.419–6.502	0.004
Poor	4.454	2.107–9.418	<0.001	3.051	1.436–6.484	0.004
Lymphovascular invasion^*^	1.502	1.255–1.798	<0.001	–	–	0.873
Nerve invasion^*^	1.319	1.085–1.604	0.005	–	–	0.907
Adjuvant chemotherapy^*^	0.745	0.610–0.911	0.004	0.625	0.505–0.775	<0.001
CEA ≥ 5 vs. > 5 ng/mL	1.287	1.060–1.563	0.011	–	–	0.455
CA19-9 ≥ 37 vs. <37 ng/mL	1.383	1.115–1.717	0.003	–	–	0.315
pT stage						
T1	1.000			1.000		
T2	0.891	0.333–2.387	0.819	1.024	0.378–2.777	0.962
T3	1.646	0.677–4.005	0.272	1.465	0.596–3.603	0.406
T4a	3.451	1.424–8.359	0.006	2.427	0.988–5.958	0.053
T4b	4.655	1.705–12.712	0.003	2.775	1.002–7.684	0.049
pN stage						
N0	1.000			1.000		
N1	1.607	1.014–2.549	0.044	1.856	1.162–2.963	0.010
N2	2.411	1.576–3.686	<0.001	2.509	1.634–3.854	<0.001
N3a	5.573	3.740–8.305	<0.001	5.373	3.582–8.061	<0.001
N3b	8.976	5.975–13.484	<0.001	9.005	5.858–13.843	<0.001

* *Present vs. absent. CEA, carcinoembryonic antigen; CA, cancer antigen*.

**Figure 2:**
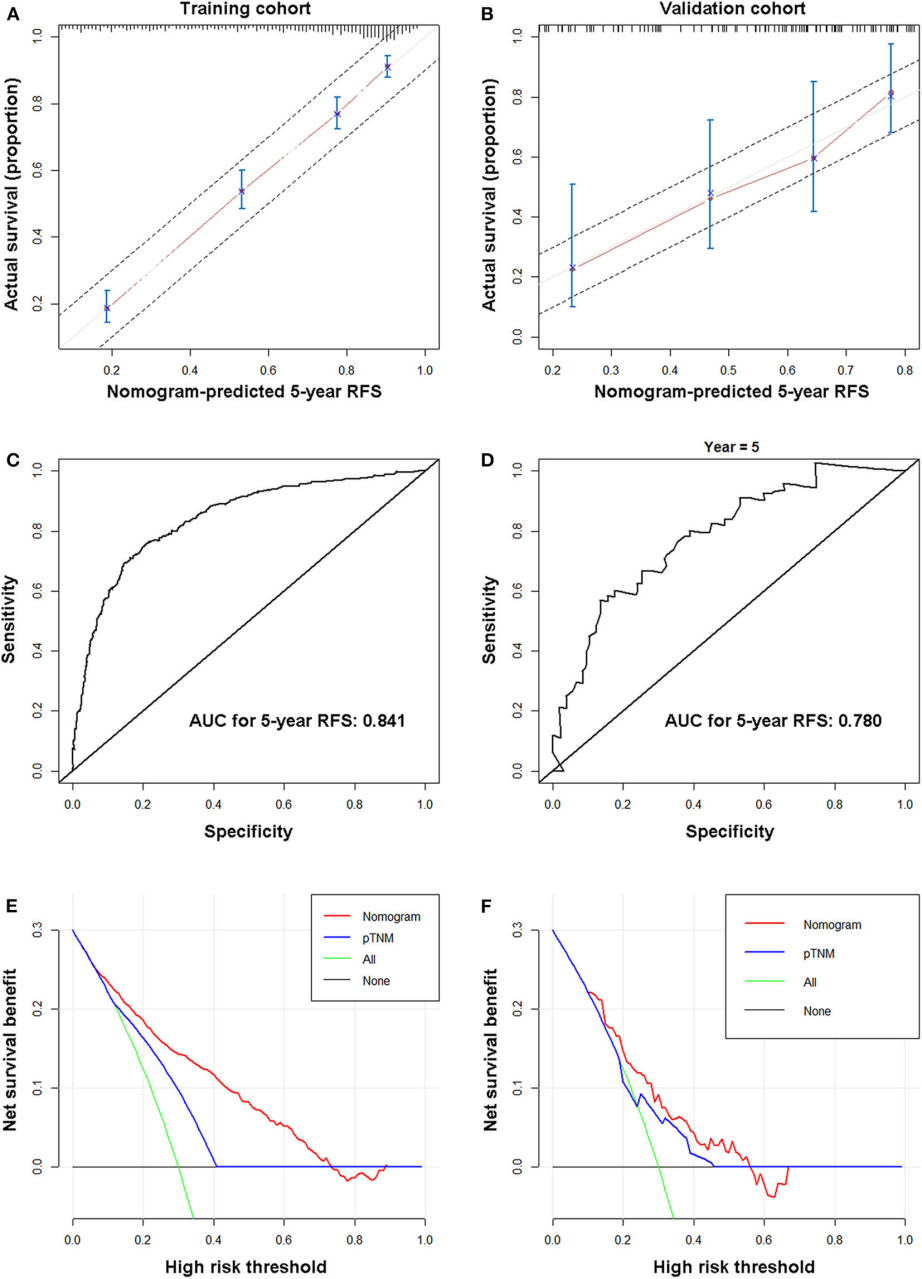
Nomogram properties. Calibration **(A,C)**, ROC curves **(B,D)**, and decision curves **(E,F)** of the nomogram for the training **(A,B,E)** and validation cohorts **(C,D,F)**.

In the training cohort, the calibration curve showed excellent agreement between nomogram-predicted and actual observed 5-year RFS (Figure 2A). Supplementary Figure 3 shows the 5-year RFS rates predicted by the nomogram of each 8^th^-AJCC-TNM classification. A wide range of predicted survival rates could be determined for each TNM stage, and patients with higher stages had a broader range of predicted survival probabilities. In addition, the model performed better than the 8^th^ AJCC-TNM in predicting 5-year RFS [C-index: 0.774 (95% CI 0.753–0.794) vs. 0.707 (95% CI 0.685–0.729), *p* < 0.001; AIC: 6,201.097 vs. 6,419.61], with a relatively high bootstrap-corrected C-index (0.774). The analysis of 5-year OS rates yielded similar results (Table 3). The ROC curve of 5-year RFS showed an excellent predictive value (AUC 0.841) (Figure 2B). The restricted cubic splines also confirmed the correlation between nomogram score and risk of recurrence (Supplementary Figure 4).

**Table 3:** Comparison of the prognostic accuracies of the nomogram and the 8^th^ AJCC-TNM.

		**Nomogram**	**AJCC 8th TNM**	***p*-value**
**Training cohort**				
RFS	C-index (95% CI)	0.774 (0.753–0.794)	0.707 (0.685–0.729)	<0.001
	AIC	6,201.097	6,419.61	–
OS	C-index (95% CI)	0.766 (0.747–0.785)	0.700 (0.680–0.620)	<0.001
	AIC	7,377.636	7,623.727	–
**Internal validation cohort**				
RFS	C-index (95% CI)	0.770 (0.730–0.810)	0.732 (0.693–0.770)	0.013
	AIC	1,249.137	1,267.904	–
OS	C-index (95% CI)	0.781 (0.742–0.821)	0.735 (0.696–0.775)	<0.001
	AIC	1,209.733	1,236.066	–
**External validation cohort**				
RFS	C-index (95% CI)	0.686 (0.609–0.763)	0.596 (0.510–0.684)	<0.001
	AIC	388.265	402.46	–
OS	C-index (95% CI)	0.692 (0.627–0.757)	0.593 (0.528–0.658)	<0.001
	AIC	604.176	627.198	–

*Statistics are for 5-year RFS and OS rates. C-index, Harrell concordance index; AIC, Akaike Information Criterion*.

In the internal validation cohort, the same findings were observed in the calibration curve (Supplementary Figure 5A). In addition, the model performed better than the 8th AJCC-TNM in predicting 5-year RFS and 5-year OS (higher C-index and smaller AIC value) (Table 3) with a high AUC for 5-year RFS (0.829) (Supplementary Figure 5B).

### Nomogram External Validation and Clinical Applicability

In the external validation cohort, the calibration curve also showed good agreement between the 5-year RFS rates predicted by the nomogram and the actual 5-year RFS rates (Figure 2C). In addition, the model performed better than the 8th AJCC-TNM in predicting 5-year RFS and 5-year OS (higher C-index and smaller AIC value) (Table 3). The ROC curve for 5-year RFS showed an excellent predictive value (AUC: 0.752) (Figure 2D).

Decision curves showed that using the nomograms to predict the 5-year RFS rates provides more benefit than the 8th AJCC-TNM in the three cohorts. (Figures 2E,F, Supplementary Figure 5C).

### Risk Group Stratification Based on Nomogram Score

**Figure 3:**
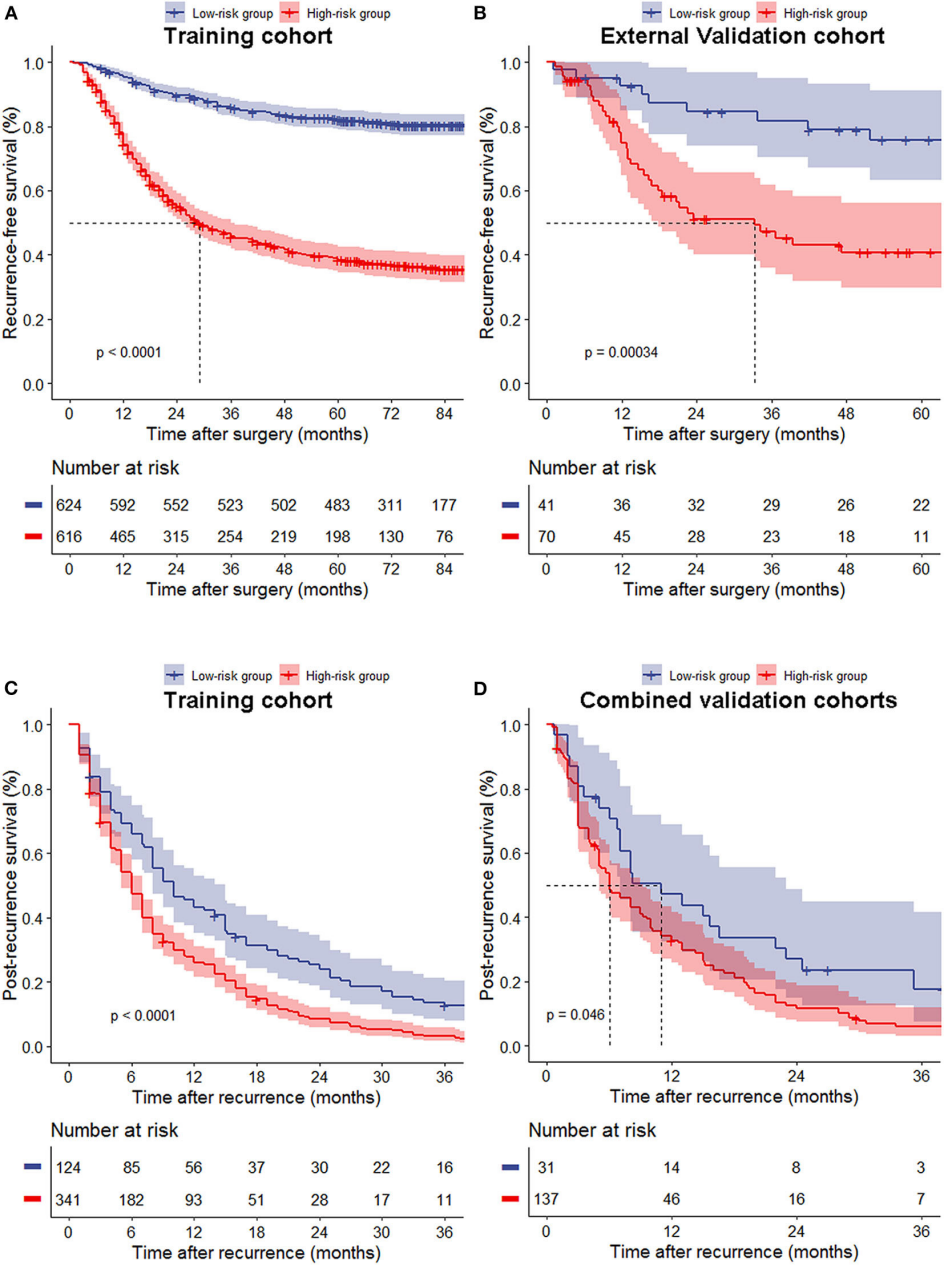
Recurrence-free survival **(A,B)** of all patients and post-recurrence survival **(C,D)** of patients with recurrence between the low- and high-risk groups in the training **(A,C)**, external validation cohort **(B)** and combined validation cohorts **(D)**.

**Figure 4:**
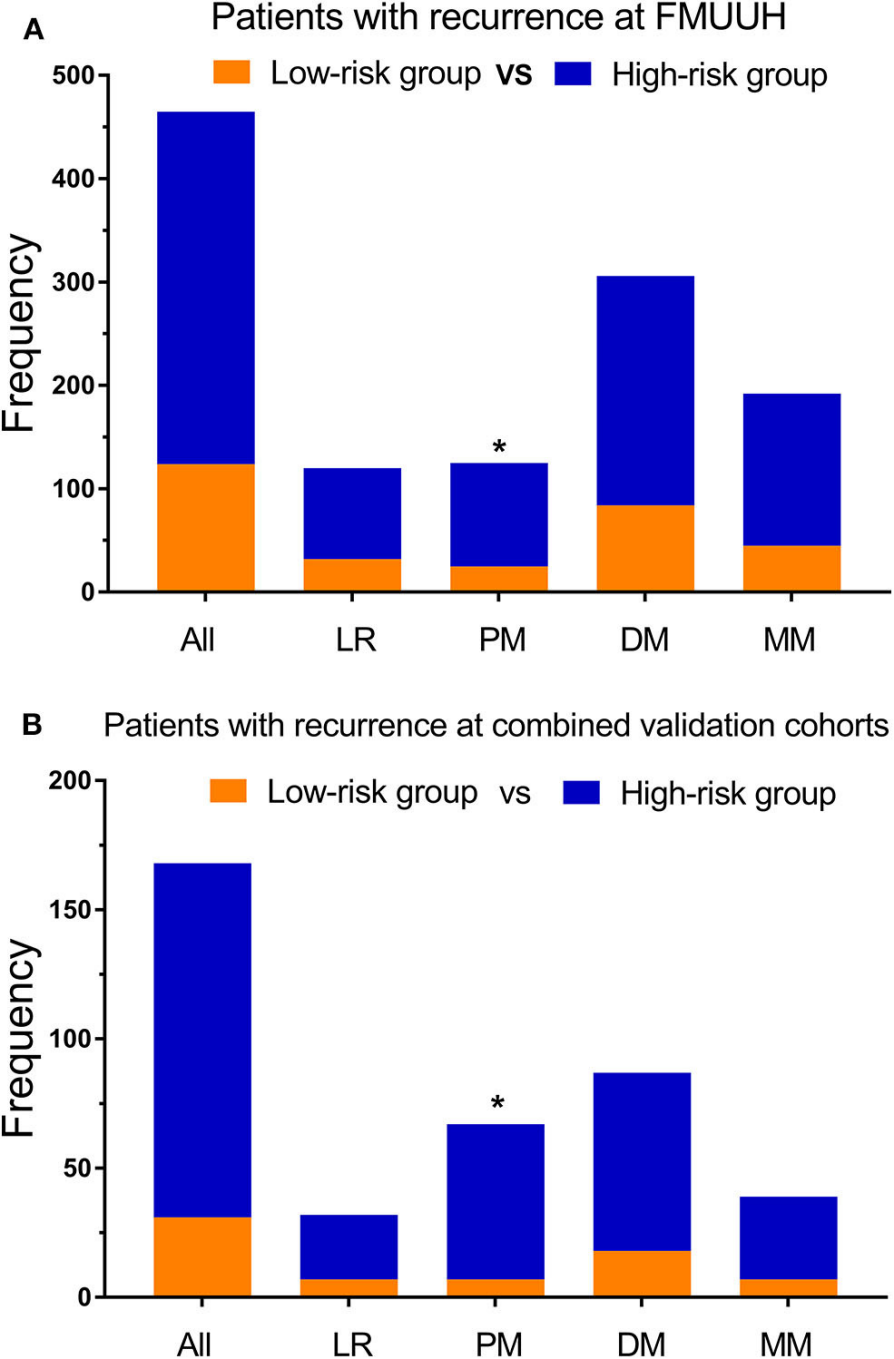
Recurrence patterns among patients determined to be at low vs. high risk using the nomogram. **(A)** Training cohort and **(B)** validation cohort. LR, locoregional recurrence; PM, peritoneal metastasis; DM, distant metastasis; MM, multiple metastasis; *p < 0.05.

The median nomogram score of the training cohort, 212, effectively distinguished populations of different recurrence risk in the training, internal and external validation cohorts (Figures 3A,B, Supplementary Figure 5D). We next analyzed the relationship between risk group and recurrence pattern in patients for whom the exact site(s) of recurrence as known [*n* = 465 for the training cohort and *n* = 168 for the combined validation cohorts (due to the small sample size of internal and external cohorts)] and found that the proportion of peritoneal metastasis was significantly higher in the high- (nomogram score >212) vs. the low-risk group (score ≤ 212) in both cohorts [29.3 vs. 20.2% and 43.8 vs. 22.6%, respectively, all *p* < 0.05], while other recurrence patterns did not significantly differ between the two groups (Figures 4A,B). Within this subset of patients (465 in the training cohort and 168 in the combined validation cohorts), the post-recurrence survival of patients at high risk was inferior to that of low-risk patients in both cohorts (Figures 3C,D).

## Discussion

The present study used data from 2010 to 2014 in a high-volume Eastern cancer center to construct a nomogram, which showed good predictive value for 5-year RFS and 5-year OS among patients with stage II/III GC. Its predictive value was also validated internally and externally using data from the U.S. In addition, the model has good predictive efficacy than the 8th AJCC-TNM. More importantly, because adjuvant chemotherapy was included in the model, it allows simple calculation of the benefit of this treatment for an individual patient. To simplify the nomogram's use, we have made it available as a free web-based calculator.

The nomogram we have developed represents an advance over other recent tools for predicting the outcomes of patients with GC after gastrectomy. Indeed, there have been several nomograms established for GC so far. The model constructed by Han et al. had significantly better predictive value for OS than the AJCC-TNM staging system (10). However, its predictive value for recurrence was not evaluated. The nomogram introduced by Jiang et al. for predicting the disease-free survival (DFS) of patients with stage II/III gastric cancer had limited predictive value, and did not include adjuvant chemotherapy as a variable despite the study's finding that it was an independent prognostic factor for DFS (16). Several scholars recommended that treatment strategy be included in a nomogram (22, 23). These two models also lacked external validation using Western data. Further, a detailed comparison with the other published predictive models (24–28) for RFS is supplemented in Supplementary Table 1. Strengths of the present study include long-term follow-up information, large sample size and patients from Eastern and Western countries.

Although the small sample size, the validation of the nomogram using Western data is a particular advantage because of the clinical and pathological differences in GC between Asia vs. the U.S. and Europe (29). For example, the proportion of diffuse-type GC is higher in Asian patients, while proximal tumors are more frequent in the West. In addition, contributing factors such as environmental exposure and diet differ between geographic regions, as do standards of treatment. Neoadjuvant therapy is preferred in the U.S. and Europe, which is not common in China. In the present study, we focused on the efficacy of postoperative adjuvant chemotherapy and only included patients without neoadjuvant therapy. The postoperative adjuvant chemotherapy consisted of either single-agent 5-fluorouracil (5-FU) or a combination of 5-FU and cisplatin/oxaliplatin or paclitaxel in both China and the U.S. It is interesting that despite the selection bias of including only Western patients who did not receive neoadjuvant therapy, who are presumably the more frail and older patients, the nomogram still demonstrated remarkable predictive value for these patients. In addition, despite the differences in background characteristics, our model still showed strong predictive power in the external validation cohort (AUC 0.780), making it widely applicable.

The ability of the nomogram developed herein to calculate the benefit of adjuvant chemotherapy represents another important step forward. While randomized clinical trials have shown improvements in DFS and OS with adjuvant capecitabine + oxaliplatin (12) and S-1 (13), their benefit for all stage II/III GC patients remains unclear. While Jiang et al. two predictive models allowed calculation of the difference in survival for the same patient if they did or did not receive chemotherapy, their predictive value was not ideal, and required two separate manual calculations (16). In addition, the fact that the model was built on data from two separate groups likely created bias, and it was not validated in a Western population. In contrast, our model was created using data from all stage II/III GC patients together, allows more straightforward calculation of chemotherapy benefit, and showed good predictive performance (C-index: 0.774; AUC: 0.841) that was validated in a Western population. In addition, the model is available as a web-based tool, which makes the calculation more easily (Supplementary Figure 2). We just type in the patient's information on the web page, the 5-year RFS probabilities both for the situation in which the patient receives or does not receive ACT are calculated automatically. The difference between the two is the net survival benefit from the addition of ACT. However, the threshold difference in RFS at which ACT provides a net benefit remains undetermined, calling for further prospective studies to identify a specific cut-off.

The current study also sheds light on postoperative recurrence patterns, which is important for developing appropriate follow-up and treatment strategies. Among the 633 patients with recurrence, those with a nomogram score >212 were more prone to peritoneal metastasis (~30%). Differences in recurrence patterns have been associated with varying pathological stage (8) and Lauren type (2), and increased risk of peritoneal metastasis has previously been linked to gastric signet ring cell histology and neural invasion (9). As the outcomes of patients with peritoneal metastasis are poor, early monitoring is essential, as is reducing its incidence. To that end, early postoperative hyper thermic intraperitoneal chemotherapy (HIPEC) has been shown to reduce peritoneal metastasis (3 vs. 23%, *p* < 0.05) (30), with similar findings in another randomized controlled trial (RCT) from Russia (31). Therefore, further RCTs determining whether early postoperative HIPEC may be appropriate treatment for patients considered at high risk for peritoneal metastasis, such as those with high nomogram scores, are warranted.

It's known that peri-operative chemotherapy for GC patients is commonly used in the West. While postoperative chemotherapy may seem redundant for patients with neoadjuvant chemotherapy, it may be beneficial, as a study by Schumacher et al. showed that patients with advanced gastric cancer who underwent surgery with neoadjuvant therapy had improved R0 resection rates, but no survival benefit (32). However, the present study and previous studies on the efficacy of adjuvant chemotherapy have excluded patients who received neoadjuvant chemotherapy, so determining its benefit in such patients requires further study.

The present study has several limitations. Many levels of selection bias could result from its retrospective nature and non-random assignment of adjuvant chemotherapy, which was based on clinician experience and patient condition. Second, due to the data limitation and to simplify the model and improve its clinical applicability, we did not explore the effect of the number of chemotherapy cycles on prognosis. Third, due to the data limitation, we only considered the survival benefit of adjuvant chemotherapy and not its side effects, which vary from person to person (33). Previous studies showed that grade 3 or 4 adverse events were reported in 10%~55% patients with different chemotherapy regimens (34–36), which were in the acceptable range. However, PAC should be withheld from patients who were identified in the no-benefit group to avoid unnecessary adverse effects related to PAC. Finally, we did not consider the impact of tumor immune-related indicators and microsatellite instability. Even though an increasing number of studies have shown them to be associated with prognosis lack of benefit of chemotherapy in gastric cancer (14, 15), they are not likely to come into common use recently.

## Conclusion

Using data from Asia and the U.S., we established and validated a predictive model that can effectively predict 5-year RFS and the benefit of adjuvant chemotherapy after radical resection of stage II/III GC. We showed that the model has significantly better predictive power and clinical applicability than the AJCC-TNM staging system. The tool is available as a simple calculator via the web, making it easier for clinicians to apply. Further large-scale, prospective, external validation is warranted.

## Data Availability Statement

The dataset analyzed for this study is available from the corresponding author on reasonable request.

## Ethics Statement

The studies involving human participants were reviewed and approved by Fujian Medical University Union Hospital Ethics Committee and Mayo Clinic Ethics Committee. The patients/participants provided their written informed consent to participate in this study.

## Author Contributions

JL, B-bX, MT, and C-mH conceived the study, analyzed the data, and drafted the manuscript. C-hZ, PL, J-wX, and J-bW helped critically revise the manuscript for important intellectual content. PL, J-wX, J-bW, J-xL, and Q-yC helped collect data and design the study. All authors contributed to the article and approved the submitted version.

## Conflict of Interest

The authors declare that the research was conducted in the absence of any commercial or financial relationships that could be construed as a potential conflict of interest.

## Supplementary Material

Supplementary Figure 1 Data collection flow chart.

Supplementary Figure 2 Web-based calculating tool that can be used to calculate an individualized estimate of 5-year RFS probabilities both with and without adjuvant chemotherapy (ACT) and then estimate the net survival benefit from the addition of ACT for patient with stage II/III gastric cancer.

Supplementary Figure 3 The box plot represents the distribution of nomogram-predicted 5-year RFS according to 8th-AJCC-TNM classification.

Supplementary Figure 4 Restricted cubic spline plots of recurrence hazard ratio vs. nomogram score.

Supplementary Figure 5 Calibration **(A)**, ROC curve **(B)**, decision curves **(C)** of the nomogram in the internal validation cohort and recurrence-free survival **(D)** for the low- and high-risk groups in the internal validation cohort.

Supplementary Table 1 Comparison of prognostic models for recurrence-free survival.
