# Supplementary figures and images for: Development and External Validation of a Nomogram to Predict Recurrence-Free Survival After R0 Resection for Stage II/III Gastric Cancer: An International Multicenter Study
Source: Front Oncol. 2020 Oct 22;10:574611. doi: 10.3389/fonc.2020.574611 (PMC7643002; doi:10.3389/fonc.2020.574611)

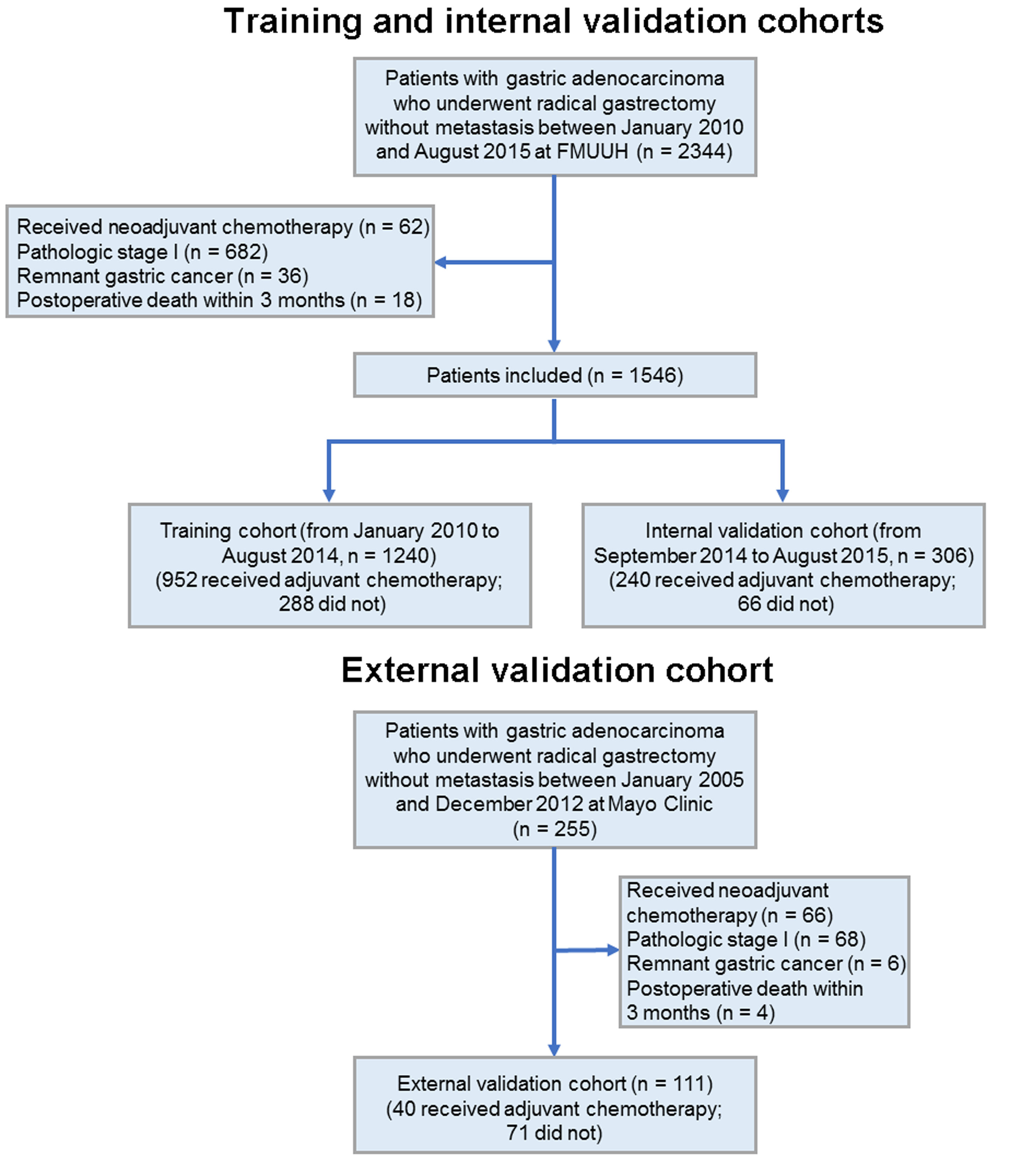

Supplement: Supplementary Figure 1 — Data collection flow chart. [file Image_1.TIF]

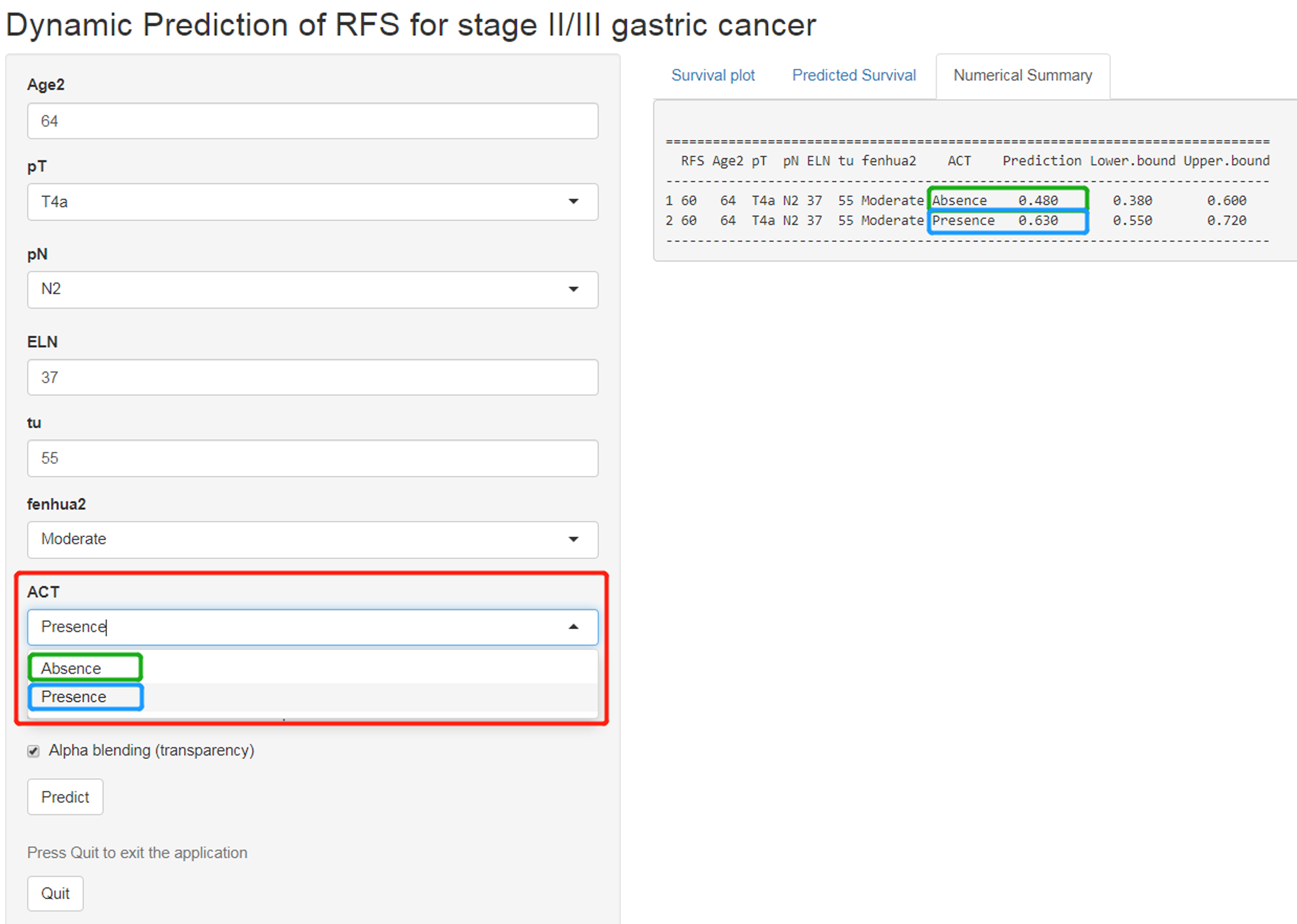

Supplement: Supplementary Figure 2 — Web-based calculating tool that can be used to calculate an individualized estimate of 5-year RFS probabilities both with and without adjuvant chemotherapy (ACT) and then estimate the net survival benefit from the addition of ACT for patient with stage II/III gastric cancer. [file Image_2.TIF]

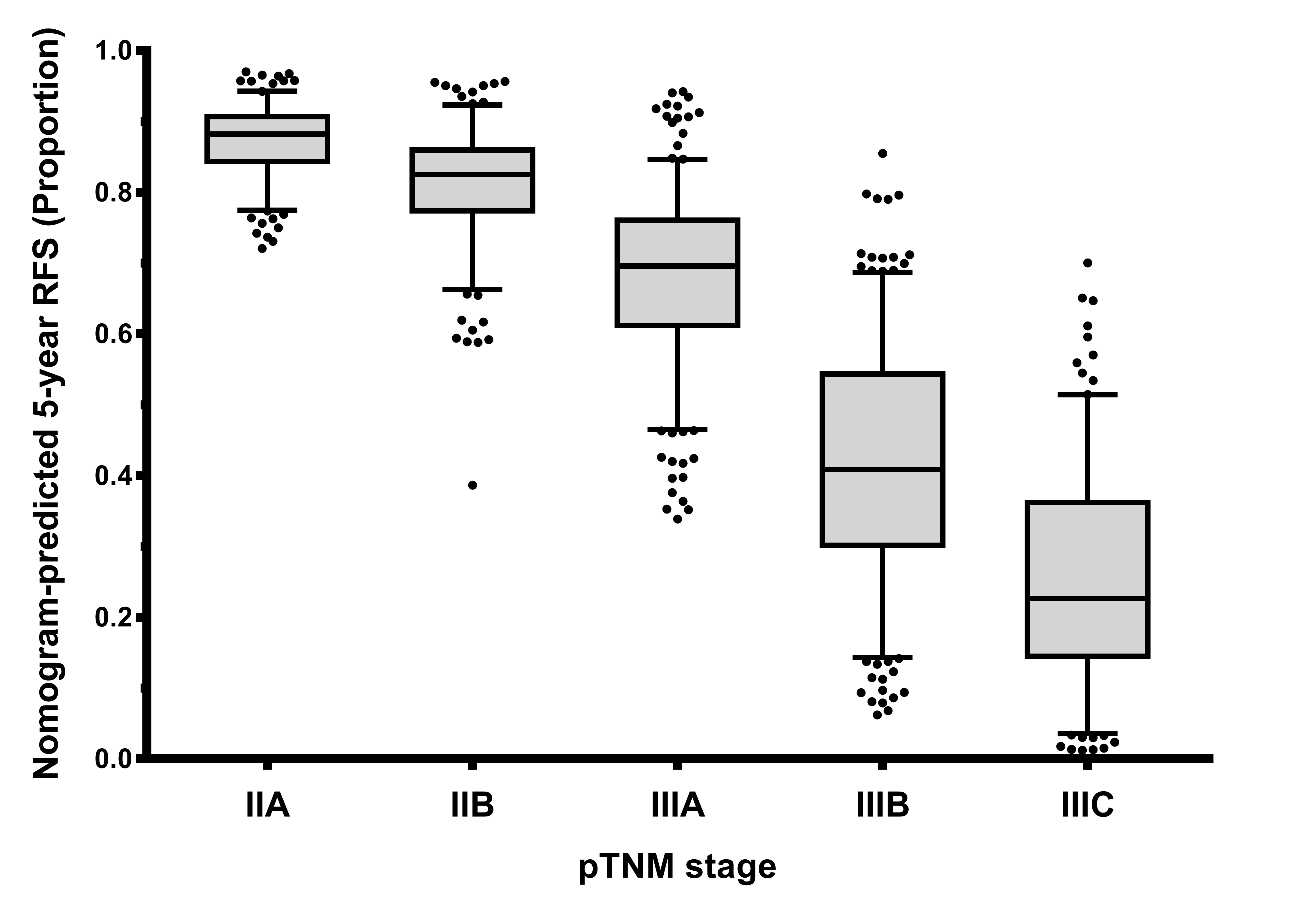

Supplement: Supplementary Figure 3 — The box plot represents the distribution of nomogram-predicted 5-year RFS according to 8th-AJCC-TNM classification. [file Image_3.TIF]

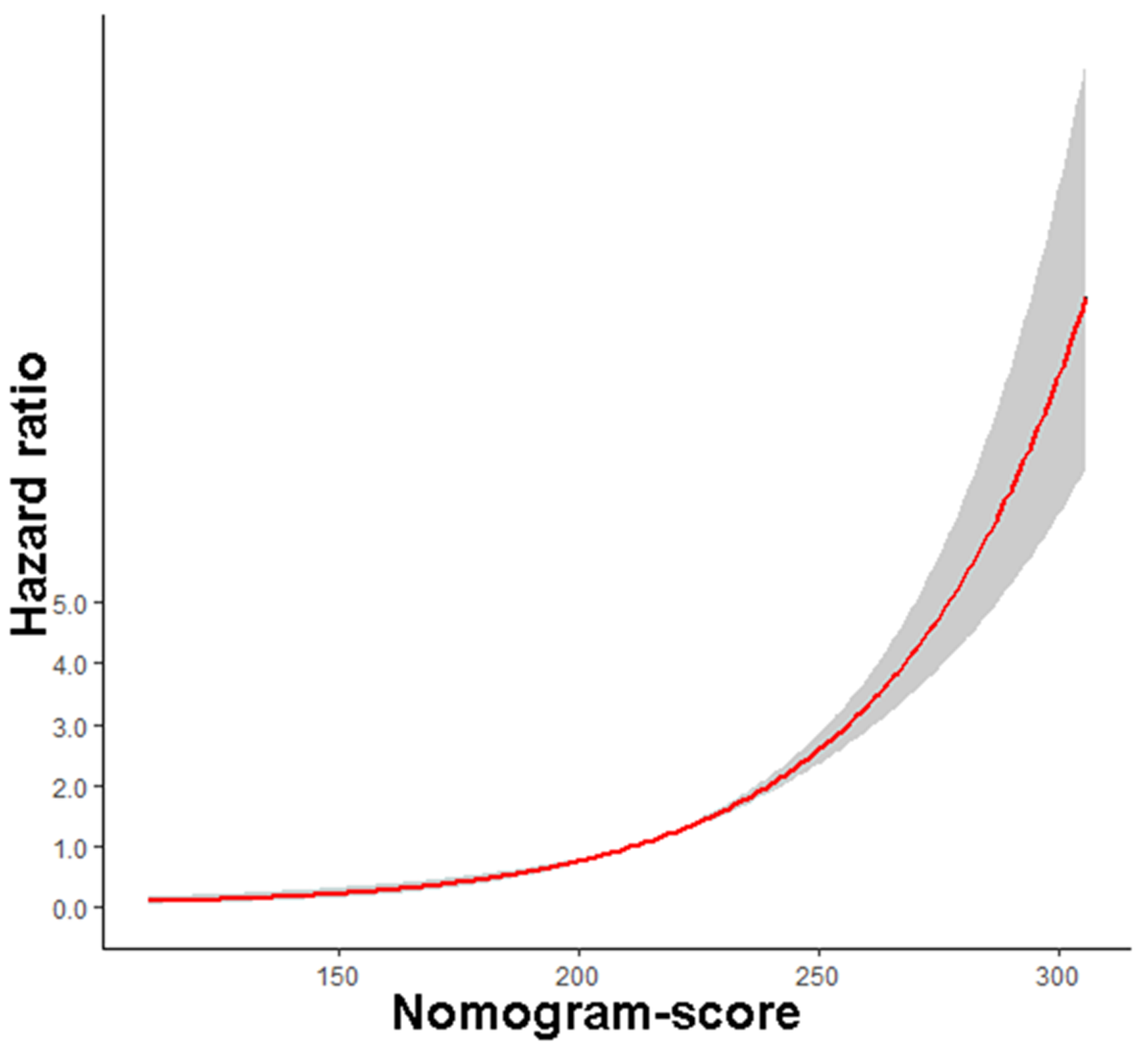

Supplement: Supplementary Figure 4 — Restricted cubic spline plots of recurrence hazard ratio vs. nomogram score. [file Image_4.TIF]

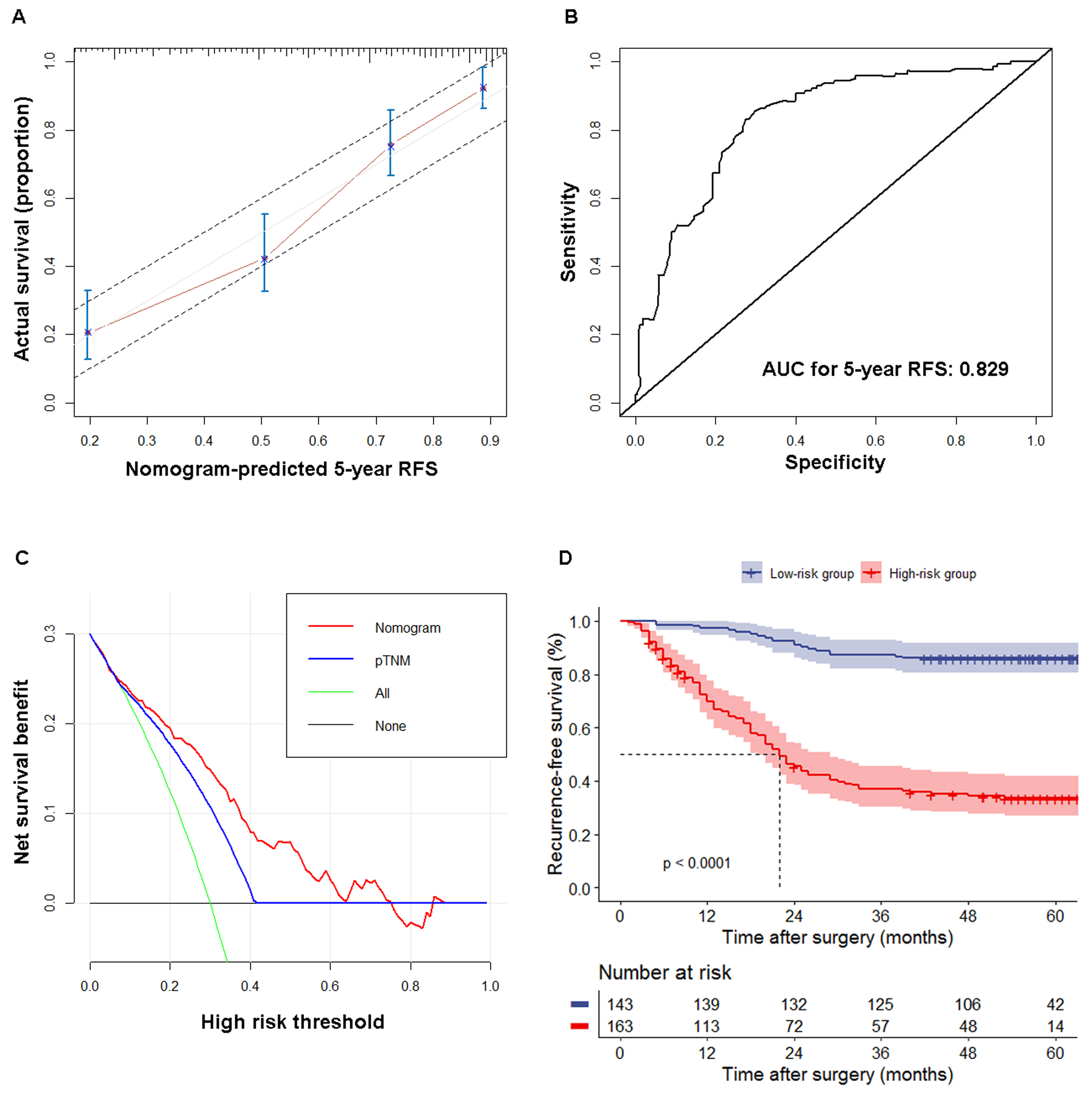

Supplement: Supplementary Figure 5 — Calibration (A), ROC curve (B), decision curves (C) of the nomogram in the internal validation cohort and recurrence-free survival (D) for the low- and high-risk groups in the internal validation cohort. [file Image_5.TIFF]
