# Supplementary material for: Development and External Validation of a Nomogram to Predict Recurrence-Free Survival After R0 Resection for Stage II/III Gastric Cancer: An International Multicenter Study
Source: Front Oncol. 2020 Oct 22;10:574611. doi: 10.3389/fonc.2020.574611 (PMC7643002; doi:10.3389/fonc.2020.574611)
Supplement: Supplementary Table 1 — Comparison of prognostic models for recurrence-free survival. [file Table_1.DOCX]

**Table S1. Comparison of prognostic models for recurrence-free survival.**

| Models | Present study | Jeong et al. [^1^](#_ENREF_1) | Wang et al. [^2^](#_ENREF_2) | Muneoka et al. [^3^](#_ENREF_3) | Lai et al. [^4^](#_ENREF_4) | Marrelli et al. [^5^](#_ENREF_5) |
| --- | --- | --- | --- | --- | --- | --- |
| Type | Nomogram | Nomogram | Nomogram | Nomogram | Nomogram | Formula |
| Year published | -- | 2019 | 2019 | 2016 | 2009 | 2005 |
| Stages included | II/III | I/II/III | I/II/III | II/III | Early (T1) | I/II/III |
| Median follow-up (months) | 79 | 60 | 28 | 75 | 62 | 56 |
| Outcome | RFS | RFS | LR | RFS | Recurrence | Recurrence |
| No. of training cohorts | 1 | 1 | 1 | 1 | 1 | 3 |
| No. of patients | 1240 | 360 | 1105 | 207 | 2880 | 658 |
| C-index | 0.774 | NA | 0.738 | 0.800 | 0.790 | NA |
| AUC | 0.841 | 0.718 | NA | NA | NA | NA |
| No. of validation cohorts | 1 | 1 | NA | NA | NA | NA |
| No. of patients | 111 | 402 | NA | NA | NA | NA |
| C-index | 0.686 | NA | NA | NA | NA | NA |
| AUC | 0.780 | 0.640 | NA | NA | NA | NA |
| Patients from Eastern and Western countries | Yes | No | No | No | Yes | Yes |
| Model variables | 7: age, tumor size, histology, examined LNs, pT, pN, ACT | 8: age, sex, expression of 6 genes | 5: age, pT, pN, CEA, lymphovascular invasion | 5: age, combined resection, pT, pN, tumor location | 9: age, sex, tumor size, gross type, histology, pT, pN, positive LNs, tumor location | 5: age, lymphadenectomy, pT, pN, tumor location |
| NA, not applicable; GA, gastric adenocarcinoma; EGC, early gastric cancer; RFS, recurrence-free survival; LR, loco-regional recurrence; DFS, disease-free survival; AUC, area under the curve; LNs, lymph nodes; ACT, adjuvant chemotherapy | | | | | | |

1. Jeong SH, Kim RB, Park SY, et al. Nomogram for predicting gastric cancer recurrence using biomarker gene expression. *Eur J Surg Oncol* 2019.

2. Wang SB, Qi WX, Chen JY, et al. Competing risk nomogram predicting initial loco-regional recurrence in gastric cancer patients after D2 gastrectomy. *Radiat Oncol* 2019; 14(1):128.

3. Muneoka Y, Akazawa K, Ishikawa T, et al. Nomogram for 5-year relapse-free survival of a patient with advanced gastric cancer after surgery. *Int J Surg* 2016; 35:153-159.

4. Lai JF, Kim S, Kim K, et al. Prediction of recurrence of early gastric cancer after curative resection. *Ann Surg Oncol* 2009; 16(7):1896-902.

5. Marrelli D, De Stefano A, de Manzoni G, et al. Prediction of recurrence after radical surgery for gastric cancer: a scoring system obtained from a prospective multicenter study. *Ann Surg* 2005; 241(2):247-55.
